# Integrase inhibitor reversal dynamics indicate unintegrated HIV-1 dna initiate *de novo* integration

**DOI:** 10.1186/s12977-015-0153-9

**Published:** 2015-03-12

**Authors:** Sylvain Thierry, Soundasse Munir, Eloïse Thierry, Frédéric Subra, Hervé Leh, Alessia Zamborlini, Dyana Saenz, David N Levy, Paul Lesbats, Ali Saïb, Vincent Parissi, Eric Poeschla, Eric Deprez, Olivier Delelis

**Affiliations:** Laboratoire de Biologie et Pharmacologie Appliquée, Centre National de la Recherche Scientifique UMR8113, ENS-Cachan, Cachan, 94235 France; Institut Universitaire d’Hématologie, Centre National de la Recherche Scientifique UMR7212-INSERM-U944, Université Paris7-Diderot, Paris, 75013 France; Laboratoire de Microbiologie Fondamentale et Pathogénicité, Centre National de la Recherche Scientifique UMR5234, Université Victor Segalen Bordeaux 2, Bordeaux, 33076 France; Department of Basic Science and Cranofacial Biology, New York University College of Dentistry, New York, NY 10010 USA; Division of Infectious Diseases, University of Colorado School of Medicine, Aurora, CO 2700 USA

**Keywords:** Integrase, Strand-transfer inhibitors, 2-LTR circles, Unintegrated viral DNA

## Abstract

**Background:**

Genomic integration, an obligate step in the HIV-1 replication cycle, is blocked by the integrase inhibitor raltegravir. A consequence is an excess of unintegrated viral DNA genomes, which undergo intramolecular ligation and accumulate as 2-LTR circles. These circularized genomes are also reliably observed in vivo in the absence of antiviral therapy and they persist in non-dividing cells. However, they have long been considered as dead-end products that are not precursors to integration and further viral propagation.

**Results:**

Here, we show that raltegravir action is reversible and that unintegrated viral DNA is integrated in the host cell genome after raltegravir removal leading to HIV-1 replication. Using quantitative PCR approach, we analyzed the consequences of reversing prolonged raltegravir-induced integration blocks. We observed, after RAL removal, a decrease of 2-LTR circles and a transient increase of linear DNA that is subsequently integrated in the host cell genome and fuel new cycles of viral replication.

**Conclusions:**

Our data highly suggest that 2-LTR circles can be used as a reserve supply of genomes for proviral integration highlighting their potential role in the overall HIV-1 replication cycle.

**Electronic supplementary material:**

The online version of this article (doi:10.1186/s12977-015-0153-9) contains supplementary material, which is available to authorized users.

## Background

Integration of the human immunodeficiency virus (HIV-1) DNA into the host cell genome is a key step in the cycle of infectious retroviral particle synthesis [1,2]. Latent HIV-1 reservoirs, such as quiescent memory CD4+ T lymphocytes, constitute the major obstacle to virus eradication during long-term antiretroviral treatment [3]. Post-integration latency probably plays the dominant role in HIV-1 persistence, but pre-integration latency, which involves unintegrated viral DNA, may also be relevant in vivo during quiescent CD4+ T cell infection, in which the virus persists as unintegrated viral DNA that is partially transcribed before cell activation [4-6]. In infected cells, including resting CD4+ T cells, unintegrated viral genomes consist of the linear form (the substrate molecule for integration generated from the reverse transcription process), circular forms resulting from autointegration and circular forms harboring one or two long terminal repeats (LTRs) (1-LTR circles: 1-LTRc and 2-LTR circles: 2-LTRc; respectively). 1-LTRc can be produced during reverse transcription as well as by homologous recombination and 2-LTRc are produced by the non-homologous end joining (NHEJ) pathway involving the ligase 4 protein [7,8]. Circularization of 2-LTRc occurs as a protective host response to the presence of linear double stranded DNA [6]. However, the nature and biological significance of the diverse forms of unintegrated molecules remain unclear in terms of their possible use as templates for transcription or as substrates for integration [9].

Regarding their relative abundance, viral DNA forms can be ranked: unintegrated linear DNA (DNA_L_) > integrated provirus (DNAi) > 1-LTRc > 2-LTRc [7]. It is important to note that the repartition of viral genomes is dynamic during the course of infection and is dependent of viral conditions of infections such as mutations in the viral proteins or addition of compounds targeting viral or cellular proteins. For example, raltegravir (RAL), belonging to the INSTI (INtegrase Strand Transfer Inhibitor) family, specifically impairs the strand transfer reaction and greatly alters the relative abundance of viral DNA species [10]. In its presence, 2-LTRc accumulate strongly due to integration inhibition, producing the same effect as integrase-disabling catalytic center mutations such as D116A [11]. It was shown that 2-LTRc represent persisting forms of unintegrated HIV-1 DNAs in non-dividing cells or in primary CD4+ T cells and are notably highly stable if cells remain growth-arrested [12-14]. They are readily detected in vivo during the natural history of HIV-1 disease in the absence of antiviral therapy and recent evidence shows they are increased in long-term elite suppressors [15]. These 2-LTRc have long been considered to be dead-end side products that do not serve as precursors to retroviral integration [16,17]. Such conclusions were drawn from experiments performed under standard condition of infection where 2-LTRc do not accumulate. Unexpectedly, integrase (IN) proteins of HIV-1 and spumaretroviruses can actually cleave the 2-LTR circle junction (which has palindromic features) and, moreover, the enzyme does so in a manner that reproduces the canonical viral CA-3’ terminus, which is needed for proper chromosomal integration and which is normally produced by IN 3’-processing of the linear cDNA [18-21]. Therefore, in the present study, we re-addressed the 2-LTRc status by investigating the consequences in cells of reversing prolonged RAL-induced HIV-1 integration blocks. We show that RAL inhibition is reversible and identify a role of 2-LTRc in the resumption of viral integration. We demonstrate that, after RAL removal, a decrease in the 2-LTRc amount leading to a linear intermediate that is subsequently followed by new integration events.

## Results

### Raltegravir action is reversible in the virological context

**Figure 1:**
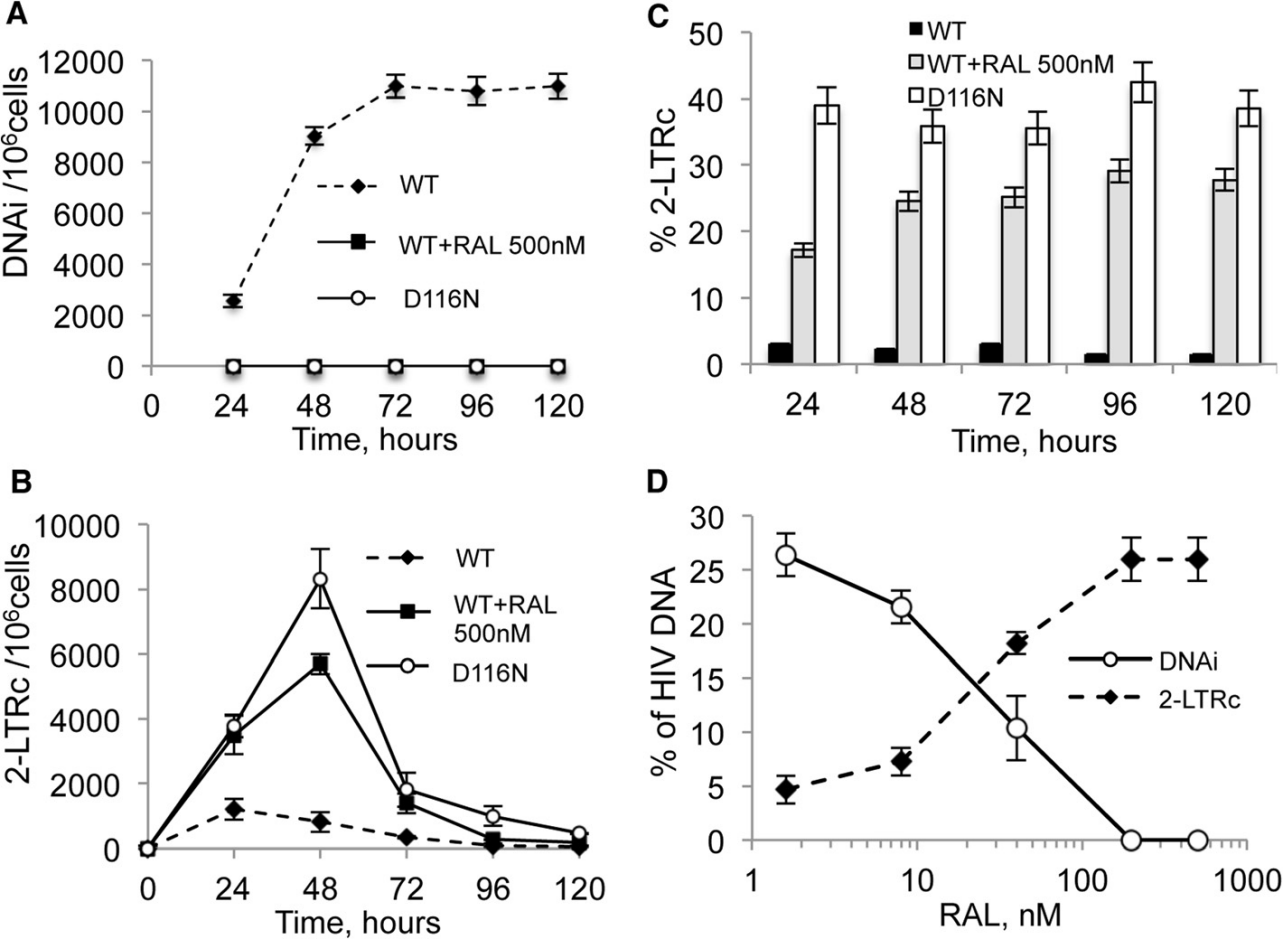
**Characterization of viral DNA forms during RAL treatment.** MT4 cells were infected with HIV-1 eGFP reporter virus (NLENG1-ES-IRES) or its D116N equivalent (p24^gag^: 40 ng/10^6^ cells). See Additional file 1: Figure S2A-B, and [24] for details about viruses. At various times after infection, real-time PCR was used to quantify: **(A)** DNAi and **(B)** 2-LTRc. **(C)** Percentages of 2-LTRc over total viral DNA. **(D)** Inverse correlation between integration inhibition and 2-LTRc formation (72 hours post-infection) in MT4 cells infected with Δenv wild-type virus in the presence of increasing RAL concentrations. Results are the mean from five representative independent experiments ± standard deviation (error bars).

RAL abolished viral replication in a standard infection assay (IC_50_ = 4 nM; Additional file 1: Figure S1), consistent with previous findings [22]. MT4 cells were infected with an envelope-pseudotyped pNL4-3(Δenv) HIV-1 reporter virus (single cycle) (Additional file 1: Figure S2A-B) in the presence or absence of 500 nM RAL. Although the sensitivity for DNAi detection in this assay is high (1 copy per 50,000 cells), no DNAi was detected when RAL was present or when a D116N (IN catalytic center mutant) HIV-1 reporter was used (Figure 1A). The integration blocks induced by RAL or the D116N mutation were accompanied by increases in both absolute levels of 2-LTRc (Figure 1B) and in their relative representativeness (i.e. the fraction of total viral DNA consisting of 2-LTRc) (Figure 1C). Furthermore, 2-LTRc accumulation correlated strongly and inversely with DNAi as drug concentration was increased (no integration was detectable at 200 nM while 2-LTRc accumulation was maximal) (Figure 1D). 2-LTRc accumulation reached 30% of the viral genome and is explained by the circularization of DNA_L_ which is more available for the NHEJ pathway when integration is inhibited [8].

**Figure 2:**
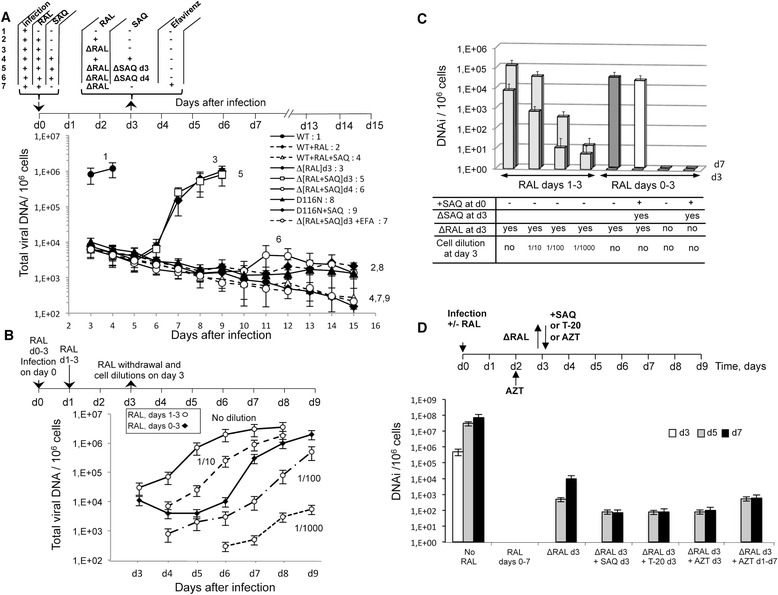
**Reversibility of RAL action.** Quantification of total viral DNA at various time post-infection **(A-B)**. **(A)** MT4 cells were infected on d0 with pNL4-3 WT or D116N (Additional file 1: Figure S2C) +/−RAL (500 nM), +/−SAQ (1 μM). On d3, RAL and/or SAQ were removed (∆RAL or ∆SAQ) or maintained (+); Efavirenz (500 nM) was added (+) or not (−) to the experiment. Black triangle, curve 8: D116N infection; black diamond, curve 9: D116N + SAQ infection. **(B)** RAL blockade from d0-3 (black diamonds) compared to blockade from d1-3 (open circles). On d3 RAL was removed from all cultures, and the RAL d1-3 culture was diluted 1/10, 1/100 or 1/1000 with uninfected cells or left undiluted. **(C)** Quantification of DNAi on d3 and d7 for the experiments shown in panels **A-B**. **(D)** Quantification of DNAi with RAL added and removed on d0 and d3, respectively. SAQ (1 μM), or T-20 (1 μM) was added on d3, when RAL was removed. AZT (25 μM) was added at d3 when RAL was removed or at d1 and maintained until d7. Results are the mean from three representative independent experiments ± standard deviation (error bars).

We then assessed reversibility of the RAL integration blockade. MT4 cells were infected on day 0 (d0) with replication-competent HIV-1 (allowing multiple rounds of infection): WT with or without 500 nM RAL, or D116N virus. On d3, RAL was either removed or maintained, and viral DNA kinetics were determined by quantitative PCR (Figure 2A). RAL caused 2-LTRc accumulation and prevented provirus formation since the amplification signal for integrated DNA obtained with or without the Alu primers was identical (Additional file 1: Figure S3), confirming the single cycle experiments of Figure 1. After 72 hours, total viral DNA levels slightly decreased in the presence of RAL (Figure 2A, curve 2) but continuously increased in untreated cells until massive cell death, reflecting the natural course of uninhibited multiple-round infection (Figure 2A, curve 1). When RAL was removed on d3, viral replication resumed, testified by the increase of total viral DNA between d5 and d6, and increased continuously until d9 (Figure 2A, curve 3). The addition of the RT-inhibitor efavirenz at the time of RAL removal (on d3) abolished DNA synthesis showing that the observed increase in total viral DNA represented new infection cycles (Figure 2A, curve 7). It is important to note that no resumption of viral replication occurs using D116N during the course of the experiment (curves 8 and 9, respectively). These data show that viral resumption after RAL removal needs the integration process. Furthermore, co-addition (on d0) and co-removal (on d3) of RAL and the HIV-1 protease inhibitor saquinavir (SAQ) produced similar results compared to those obtained with RAL alone removed at d3 (compare curves 3 and 5 in Figure 2A). However, co-addition (on d0) and co-removal (on d4) of RAL and SAQ did not result in resumption of viral replication (curve 6). When RAL is maintained in association with SAQ, resumption of viral replication does not occur (Figure 2A, curve 4). Thus, the viral nucleoprotein complex responsible for the resumption of viral replication after RAL removal was provided by the initial infection on d0 and is still present at d3 but not at d4, suggesting that undetectable integrated DNA, if any, is not involved in this process.

### Nature of the viral genome accounting for RAL reversibility and resumption of viral replication

Two hypotheses can be formulated to explain the observed resumption of viral replication after RAL removal. First, small amounts of DNAi that are undetectable by real-time PCR may have been present, or, second, *de novo* integration may have initiated from accumulated unintegrated DNA. We investigated the possible role of undetectable DNAi in resumption of viral replication, by adding RAL to MT4 cells from days 1–3 (d1-3) instead of days 0–3 (d0-3), thus allowing a 24 hour window for integration to occur. For this d1-3 RAL treatment condition (Figure 2B, open circles), three parallel cultures were also set up at the time of drug removal, on d3, by diluting infected cells with uninfected cells at ratios of 1:10, 1:100 and 1:1000. As expected, viral replication was dose-dependent on the presence of DNAi. The kinetics of viral replication (indicated by the measure of total viral DNA amount) in cells treated with RAL from d0-3 (black diamonds) was more rapid than those of the 100- and 1000-fold dilution (d1-3 RAL) cultures (Figure 2B). Interestingly, this was the case even though no DNAi was detectable on d3 in the d0-3 RAL culture, whereas DNAi was easily detectable at d3 in the d1-3 RAL culture, regardless of the dilution factor (even in the 1:1000 dilution culture) (Figure 2C). Thus, undetectable DNAi generated up to d3 in the d0-3 RAL culture cannot account for the kinetics of replication observed after RAL removal. These results revealed that when RAL blockade is relieved after 3 days, the source of resumed HIV-1 replication is unintegrated DNA which is further used for *de novo* integration. These data exclude a major role of undetected integrated DNA in viral resumption after RAL removal.

Indeed, RAL removal was associated with a significant increase in integration events by d5 in d0-3 RAL blockade cultures (Figure 2C-D) and a production of infectious viral particles (Additional file 1: Figure S4). Integration events detected on d5 occurred whether or not drugs that prevent successive infection rounds (SAQ, T-20 or AZT) were added at the time of RAL removal on d3. Thus, they fully reflected *de novo* integration arising from pre-accumulated unintegrated viral DNA originating from infection at d0 and still present at d3. A last condition was performed adding RAL at d0 and AZT at d1 (AZT was maintained until d7), allowing reverse transcription to occur but preventing a weak replication from unintegrated viral DNA as highlighted by Trinite and colleagues [23]. In this condition, when RAL was removed at d3, the amount of DNAi at d5 was similar to the one quantified in the condition without AZT, excluding a major role of the replication from unintegrated viral DNA in the detection of new integration events after RAL removal (Figure 2D). In contrast, the further increase in DNAi on d7 (compared to d5) in the absence of SAQ, T-20 or AZT reflected subsequent rounds of infection (Figure 2D). Newly integration events are thus compatible with synthesis of new viral progeny highlighting that integration from pre-accumulated unintegrated viral DNA is biologically relevant.

### Newly integration events after RAL removal result from a DNA_L_ intermediate generated from 2-LTRc

**Figure 3:**
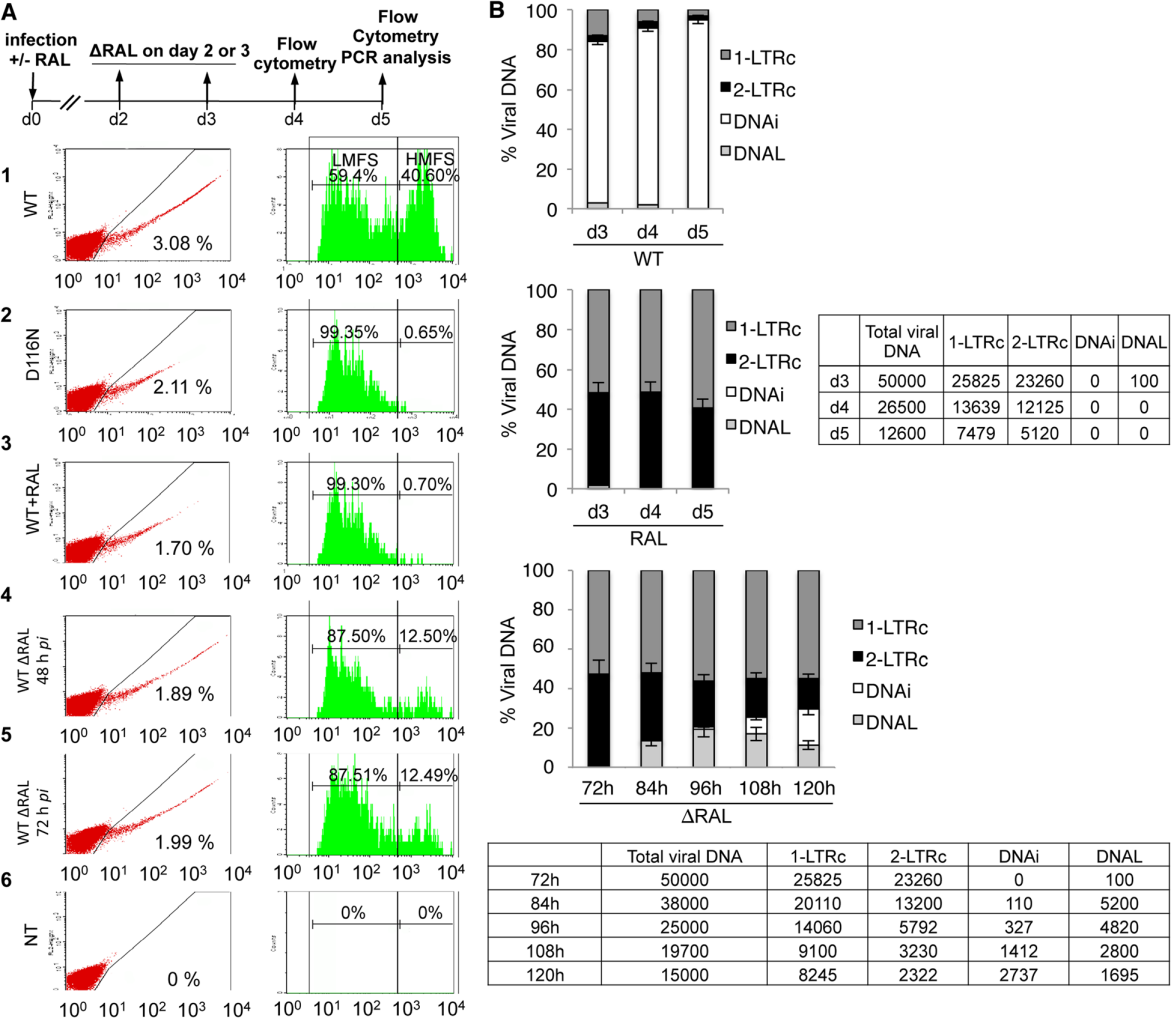
**2-LTR circles account for** ***de novo*** **integration and the resumption of viral replication after RAL removal. (A)** Flow cytometry analysis of envelope-pseudotyped viruses NLENG1-ES-IRES expressing eGFP (Additional file 1: Figure S2A-B). **1**: WT without RAL. **2**: D116N without RAL. **3**–**5**: WT + 500 nM RAL (added at d0). RAL was maintained (**3**) or removed on d2 (**4**) or d3 (**5**). **6**: NT: non-transduced cells. We monitored eGFP expression on d4 (**4**) or d5 (**1**–**3**, **5**–**6**). Left, percentage of GFP+ cells. Right, gating on the GFP+ cells to discriminate DNAi expression (HMFS: high mean fluorescence signal) from unintegrated viral DNA expression (LMFS: low mean fluorescence signal). Data are representative of five independent experiments. **(B)** Quantification of DNAi, 2-LTRc, 1-LTRc and DNA_L_ corresponding to experiments **1**, **3** and **5**. RAL was removed (ΔRAL) or maintained (RAL) at d3 post-infection. Percentage of 1-LTRc (dark grey column), 2-LTRc (black column), percentage of DNAi (white column) and percentage of DNA_L_ (light grey column) are calculated. Raw data in copy number are reported. Results are the mean from five representative independent experiments ± standard deviation (error bars).

To confirm that the *de novo* integration events originate strictly from accumulated unintegrated DNA forms, we then assessed RAL reversal using single cycle eGFP reporter viruses that prevent successive rounds of infection [24]. Cells were infected at low multiplicity of infection (m.o.i.) (Figure 3, 40 ng of p24^Gag^ per 10^6^ cells) or at a higher m.o.i. (200 ng of p24^Gag^ per 10^6^ cells, Additional file 1: Figure S5). Cell fluorescence intensities had bimodal distributions (Figure 3A, right). High mean fluorescence signal (HMFS) originates from the strong transcriptional activity of DNAi, whereas the low mean fluorescence signal (LMFS) originates from the weaker transcriptional activity of unintegrated DNA as already described [24]. In the absence of RAL (WT), 3.08% of the total cell population was GFP+. 59.4% of the GFP+ cells displayed LMFS and 40.6% displayed HMFS (Figure 3A, panel 1). In contrast, HMFS was negligible when integration was prevented: LMFS was 99.3% with RAL treatment or when a D116N mutant reporter virus was used (Figure 3A, panels 2–3). Upon RAL removal, HMFS increased from 0.7% to 12.5%, reflecting *de novo* integration under both conditions, 48 and 72 hours post-infection (Figure 3A, panels 4–5). RAL removal at 72 hours post-infection from infected cells with a higher m.o.i. led to an increase of HMFS from 1.1% to 19.3% (Additional file 1: Figure S5A). Accordingly, IN was still detected 72 hours post-infection in RAL-treated cells (Additional file 1: Figure S6) suggesting the role of IN in this process. DNAi and 2-LTRc quantifications confirmed that integration blockade by RAL treatment was complete. Indeed, no DNAi was detected and this absence was correlated with 2-LTRc accumulation compared to the condition without RAL (Figure 3B, upper and middle panels).

To gain insight into the mechanisms of RAL reversal, we quantified in an exhaustive manner all viral DNA forms: 2-LTRc, 1-LTRc, DNAi and DNA_L_ based on previous reports [7]. More particularly, DNA_L_ was quantified using a linker-mediated PCR approach [7]. Briefly, a linker compatible to the 3’-processed end of the linear DNA, was used. This linker was able to ligate to both unprocessed and 3’-processed DNA. After ligation, the DNA was purified and quantified using quantitative PCR with primers hybridizing in the linker and in the LTR. RAL treatment led to a strong circular viral forms accumulation (1-LTRc and 2-LTRc) where the 2-LTRc representativeness reached 45% of total viral DNA at d3 post-infection (Figure 3B, middle panel, black column). No DNAi was detected when cells were treated with RAL as previously described (Figure 3B, middle panel, white column). At d3 post-infection, when RAL is removed, DNA_L_ represented 100 copies i.e. 0.2% of total viral DNA (Figure 3B, middle and bottom panels). Most importantly, from d3 to d5 post-infection, after RAL removal, we consistently observed a transient and significant increase in DNA_L_ reaching 20% of total viral DNA concomitant with a decrease of 2-LTRc and increase of DNAi at d5 post-infection (Figure 3B, bottom panel). The anti-correlation between the decrease of 2-LTRc and DNAi was also observed at higher m.o.i using RAL or Elvitegravir (EVG), another strand-transfer inhibitor (Additional file 1: Figure S5B and C). When RAL was maintained, no DNA_L_ or DNAi was detected at d4 or d5 post-infection (Figure 3B, middle panel) and no decrease of 2-LTRc percentage is observed. Since RAL does not influence cell division, the fact that the representativeness of 2-LTRc was not changed when RAL was maintained highlights that the 2-LTRc decrease observed when RAL was removed is not due to cell division. Furthermore, when RAL was removed, the increase in the amount of DNA_L_ was only transient since DNA_L_ further decreased concomitant to the observed increase in DNAi. To note, the 1-LTRc amount did not vary significantly between d3 and d5 post-infection (when RAL was maintained or after RAL removal) suggesting that 1-LTRc did not play important role in the observation of neo-integration events.

Taken together, these data demonstrate that RAL removal led to a decrease of 2-LTRc leading to an increase of DNA_L_ that is integrated into the host cell genome. Quantifications of viral DNA genomes at d3, d4 and d5 demonstrate that, even if unintegrated DNA (linear and circular) are diluted due to cell division (from d3 to d5), amount of linear DNA (0.2% of the viral genome i.e. 100 copies) at d3 post-infection does not account for the amount of integrated viral DNA at d5 (18% of the viral genome i.e. 2,737 copies) (Figure 3B). In combination with the reciprocal correlation between 2-LTRc and DNAi, the re-appearance of DNA_L_ at d4 after RAL removal and its further decrease (at d5) when DNAi increases suggests that the resumption of viral replication originates from integration of newly generated DNA_L_ derived from 2-LTRc. A major role of DNA_L_, provided from the initial infection (d0) after reverse transcription, in the recovery of integration events can then be excluded.

Experiments were also performed with CD4+ primary cells in the same conditions as described for MT4 cells except that the m.o.i. was increased. Again, RAL reversibility was observed when RAL was removed at 48 or 72 hours post-infection (Additional file 1: Figure S7A): Upon RAL removal, HMFS increased demonstrating eGFP expression from newly integrated viral DNA. Quantitative PCR demonstrates that RAL removal, as described for MT4, results in a decrease in 2-LTRc (Additional file 1: Figure S7B, middle panel) correlated by detection of new integration events (Additional file 1: Figure S7B, upper panel). Indeed, integration recovery was accompanied by a 2-fold decrease in 2-LTRc, consistent with an interpretation that 2-LTRc is used as precursors for integration (Additional file 1: Figure S7B, lower panel). These data indicate that RAL action is also reversible in primary cells infection.

### The linearization of 2-LTRc requires the integrity of catalytic activity of IN

**Figure 4:**
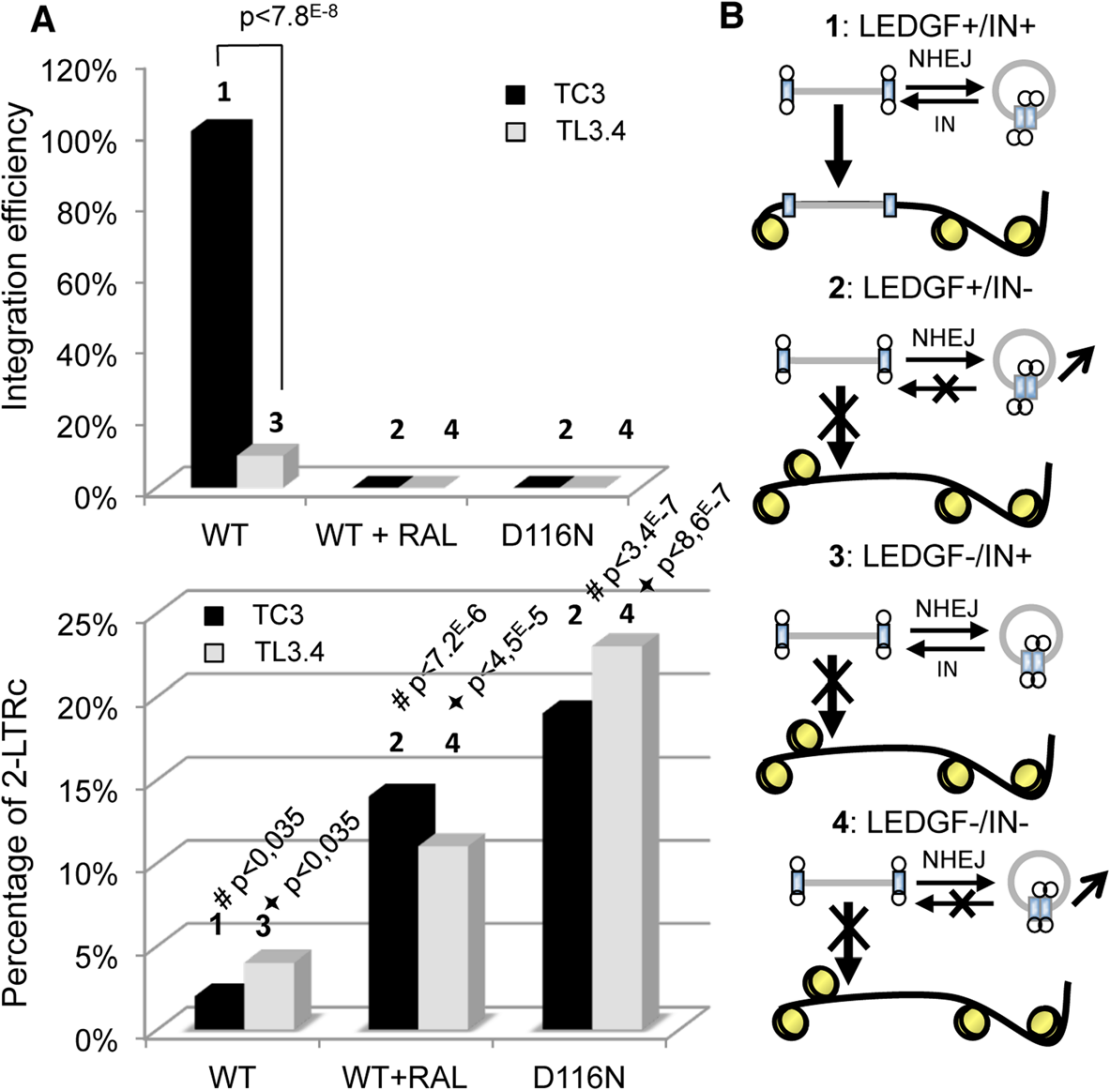
**2-LTRc are related to the IN catalytic activity.** Cells expressing (TC3) or depleted (TL3.4) of LEDGF/p75 were infected with NLENG1-ES-IRES-WT +/− 500 nM RAL or NLENG1-ES-IRES-D116N. **(A)** Percentage of integration efficiency (upper panel) and 2-LTRc (lower panel) at d3 post-infection for both cell lines. Results are representative of four independent experiments; p value is reported in the histogram. **(B)** Model explaining the modulation of 2-LTRc amount in different contexts: LEDGF+ (TC3) or LEDGF- (TL3.4) with IN+ (WT) or IN- (D116N or WT + RAL). The numbers above the bars in panel **A** are related to the corresponding model in panel **B**.

To determine whether the IN catalytic activity is required for the 2-LTRc - > DNA_L_ conversion, we performed experiments under conditions where integration is inhibited while IN enzyme function is preserved. In addition to RAL treatment and mutations such as D116N, viral integration can be inhibited by making cells deficient in the integration cofactor LEDGF/p75 [25,26]. We infected TC3 and TL3.4 cells, which are human SupT1 CD4+ T cells that express a control shRNA and a highly effective LEDGF/p75-targeting shRNA, respectively [26]. TL3.4 cells infected with WT HIV-1 (without RAL) exhibited a major decrease in integration as expected (7-fold) (Figure 4A, upper panel). However, this decrease was associated with only a slight increase in the amount of 2-LTRc in these cells (2-fold) (Figure 4A, lower panel). This phenomenon represents an experimental case in which integration inhibition does not systematically lead to strong 2-LTRc accumulation. Indeed, in the TL3.4 cells, 2-LTRc accumulation was observed only with RAL treatment or D116N (Figure 4A, lower panel). This also suggests that LEDGF/p75 is not an essential factor for 2-LTRc formation. It is important to note that the inhibition of the remaining integration between conditions of TL3.4 infected with WT, and TL3.4 infected with D116N or in the presence of RAL (Figure 4A, upper panel, columns 3 and 4), cannot explain the difference of 2-LTRc accumulation in these conditions of infection (Figure 4A, lower panel, columns 3 and 4). Indeed, the observed 7-fold decrease in integration is expected to display 85% of maximal 2-LTRc accumulation based on the correlation shown in Figure 1D. Moreover, as described previously in other cell lines, RAL reversal produced a fall in the percentage of 2-LTR circles in both cell lines (Additional file 1: Figure S8).

These results indicate that when integration is inhibited without blocking IN catalytic competence (i.e. in the absence of LEDGF), the inhibition is not necessarily associated with 2-LTRc accumulation since IN is still competent to cleave 2-LTRc (see model in Figure 4B). Accordingly, inhibiting the catalytic activity of IN (RAL treatment or D116N infection) leads to accumulation of 2-LTRc due to the inability of IN to cleave these circular DNA forms. Our data imply that IN plays a key role in controlling the balance between the amounts of DNA_L_ and 2-LTRc through direct effects on 2-LTRc - > DNA_L_ conversion. In this context, RAL removal leads to 2-LTR_C_ cleavage, which in turn produces new DNA_L_ that can integrate and support resumption of viral replication. Taken together with the above-mentioned observation of new DNA_L_ forms after RAL removal, our data indicate that IN catalytic activity is directly involved in the 2-LTRc - > DNA_L_ conversion.

### Integrase is found at the palindromic junction of 2-LTRc

**Figure 5:**
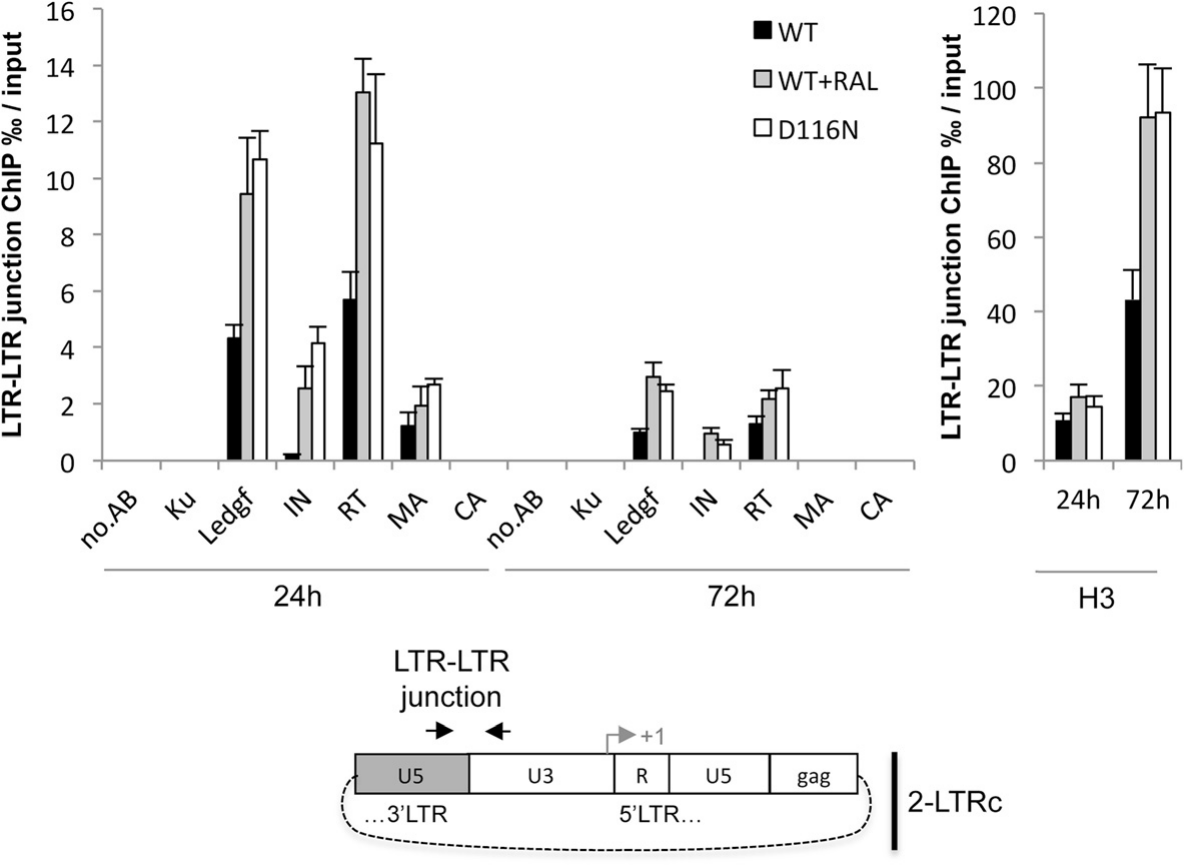
**Integrase and other viral and cellular partners are present at the LTR-LTR junction.** MT4 cells were infected with NLENG1-ES-IRES-WT in absence (black bars) or in presence of 500 nM RAL (grey bars), or infected with NLENG1-ES-IRES-D116N (white bars). ChIP experiments were conducted without antibodies (NO.AB) or with antibodies against histone H3 (H3), Ku-80 (Ku), LEDGF/p75 (LEDGF), HIV-1-integrase (IN), HIV-1-Reverse-Transcriptase (RT), HIV-1-p24-capside (CA) or HIV-1-matrix-structural-protein (MA) (top) at 24 and 72 hours post-infection. Real-time PCR was used to analyze the nature of the DNA bound to proteins at the LTR-LTR junction (+/−200 bp) (bottom). Arrows highlight the positions of the primers used for quantitative PCR. Results are the mean from three representative independent experiments ± standard deviation (error bars).

The IN-dependent conversion of 2-LTR circles into DNA_L_ implies that IN should be physically present at the palindromic junction. It has been demonstrated by Bukrinsky and collaborators that HIV-1 IN was not detected in association with 2-LTRc [27]. Importantly, this result was obtained under condition where no 2-LTRc accumulation was observed (i.e. WT infection in the absence of any INSTI compound). We then performed ChIP (chromatin immunoprecipitation) experiments to assess the presence of IN on 2-LTRc within cells under 2-LTRc accumulation conditions at 24 and 72 hours post-infection. According to Bukrinsky’s study [27], IN was not found at the palindromic junction during WT infection (Figure 5), probably due to the small amount of 2-LTRc present during WT/RAL- infection. However, IN was present in the region spanning the LTR-LTR junction (+/-200 bp), only under infection conditions leading to 2-LTRc accumulation (WT + RAL or D116N) (Figure 5). Interestingly, no IN was detected in a region near the +1 transcription start site of the 5’-LTR (separated by 450 bp of the LTR-LTR junction), regardless of the conditions (Additional file 1: Figure S9). These results indicate IN binding to the LTR-LTR junction during viral infection, compatible with a role of IN in cleaving this junction. The presence, at the LTR-LTR junction, of other HIV-1 (Matrix (MA), reverse transcriptase (RT), Capsid (CA)) or cellular (Ku, LEDGF) proteins, described as functionally/physically interacting with IN, was also tested. Among these proteins, MA, RT (but not CA) and LEDGF/p75 (but not Ku) were detected at the LTR-LTR junction together with IN (Figure 5). All the detected proteins have been described as belonging to the pre-integration complex (PIC) [28,29]. Moreover, H3 histones were found at both the LTR-LTR junction (Figure 5) and at the +1 transcription start site (Additional file 1: Figure S9), confirming and extending the results of the chromatin organization of unintegrated viral DNA forms as already reported [30]. Altogether, (i) the IN-dependent linearization of 2-LTRc compatible with the presence of IN as well as other PIC components at the LTR-LTR junction, and (ii) the presence of the main tethering factor for integration (LEDGF) at the LTR-LTR junction, raise the question of whether the resulting 2-LTRc cleavage product leads to correct IN dependent integration. The fact that IN was found at the palindromic junction and at d3 post-infection reinforces the hypothesis that IN is involved in the 2-LTRc - > DNA_L_ conversion.

### The pre-integration complex (PIC) is able to cleave and integrate 2-LTRc

**Figure 6:**
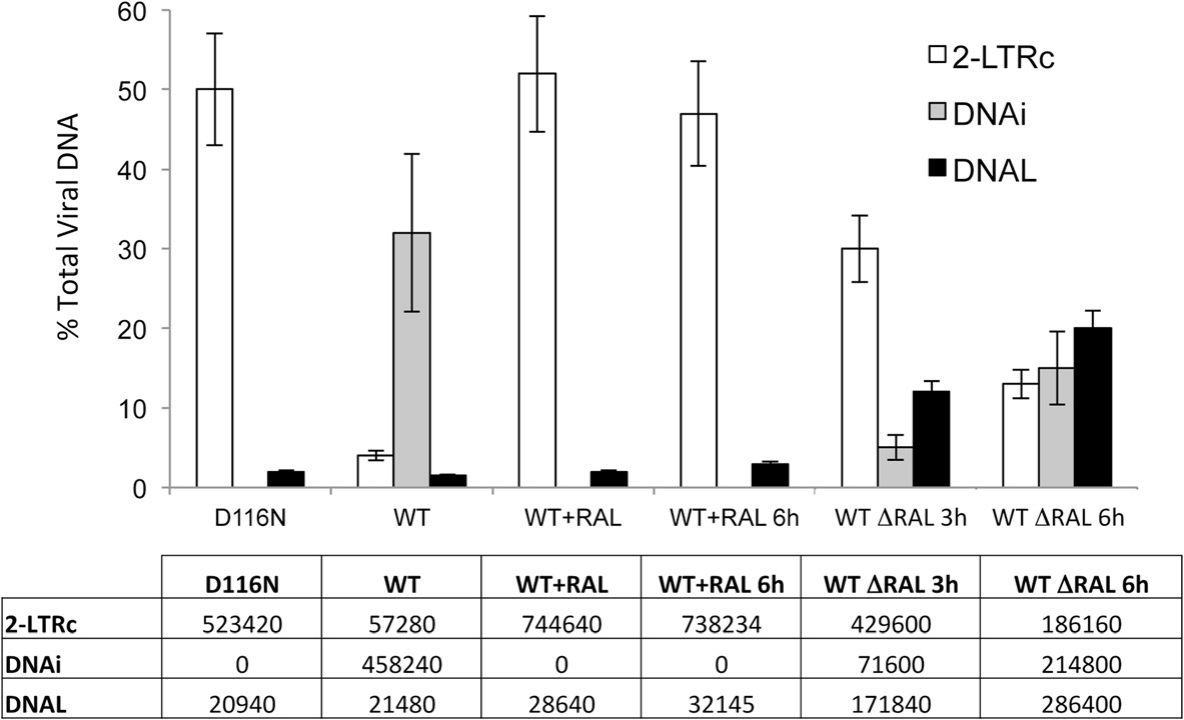
**Pre-Integration complex (PIC) is able to cleave and integrate 2-LTRc.** MT4 cells were infected with NLENG1-ES-IRES-D116N or NLENG1-ES-IRES-WT +/− 500 nM RAL. 72 hours post-infection, PIC were extracted as described previously [31]. Viral DNA genomes (2-LTRc: white column, DNAi: grey column and DNA_L_: black column) were quantified before dialysis (D116N; WT; WT + RAL) and after 3 hours (WT ΔRAL) and 6 hours of dialysis (WT ΔRAL; WT + RAL). Results are the mean from three representative independent experiments ± standard deviation (error bars).

PICs are large nucleoprotein complexes that contain several cellular and viral proteins allowing integration during infection. PIC were extracted from whole cell extract as previously reported [31] at 72 hours post-infection in order to allow the purification of 2-LTRc complexed with PIC/intasome proteins, as already observed by ChIPs experiments and to minimize PIC/intasome complexed with linear viral DNA. PIC extraction was performed from MT4 cells previously infected with NLENG1-ES-IRES-D116N or NLENG1-ES-IRES-WT +/− 500 nM RAL. Viral DNA genomes (2-LTRc, DNAi and DNA_L_) were quantified (Figure 6). As described in previous experiments, 2-LTRc were highly accumulated in D116N or RAL conditions (nearly 50% of total viral genome) and DNA_L_ is quite negligible representing 2% of the total viral genome. PICs were then submitted to dialysis for 3 and 6 hours in order to remove RAL from the PIC complex and allow integration reaction to occur. As described in cellular experiments where RAL was removed from cell medium, we observed a consistent decrease in the amount of 2-LTRc correlated with a recovery of DNA_L_ and DNAi. Importantly, such decrease of DNA_L_ and DNAi is not observed when RAL is maintained during dialysis. Interestingly, the addition of DNAi and DNA_L_ percentages (15% and 20%, respectively) corresponds roughly to the decrease in the 2-LTRc percentage (38%). This quantitative analysis demonstrates that, upon RAL removal, 2-LTRc are efficiently converted by the PIC into a linear DNA which in turn is involved in the integration process in the host cell genome. Taken together, our data show that PIC can be inhibited by RAL in a reversible manner and that 2-LTRc, accumulated under RAL treatment, can be used as substrates for integration. Taken together, the PIC experiments demonstrate that the palindromic junction is efficiently used in the integration process.

## Discussion

2-LTRc accumulate in HIV-1-infected cells *in vitro* and in vivo under a variety of conditions, including but not limited to the potent disruption of integrase catalysis caused by RAL. It is generally described that formation of these circular genomes prevents generation of apoptotic signals originating from DNA_L_ extremities. We propose that HIV-1 may utilize these ligated genomes rather than consign them to uselessness. The first one is transcription of the circular forms that can be effective in some circumstances [23,32]. However, in this study, we exclude the role of unintegrated viral DNA in viral transcription leading to viral production. Indeed, we do not observe viral replication when RAL is maintained or when a D116N virus is used. The second strategy, supported by the present data, is to cleave the ligated circle such that it can be chromosomally integrated in the host DNA and therefore represents the main way to account for RAL reversibility. Patients taking integrase inhibitors as part of therapy are unlikely to stop treatment. Other studies are needed to highlight a role of 2-LTR circles after RAL removal in the viral resumption. However, our data reinforce the fact that RAL must be maintained in the treatment and not interrupted. This reversibility may be responsible for the observed failure of intermittent antiretroviral treatments occurring only when RAL was included in combination with other drugs, without RAL resistance mutation detected in integrase [33]. Indeed, HIV “blips” (intermittent episodes of detectable low levels of HIV viremia) and virological failure were observed, for instance, when the NRTI pair (tenofovir + emtricitabine) was combined with RAL, this failure not being observed with any other drug cocktails in the absence of RAL. Our study suggests that this virological failure may be due to *de novo* integration occurring after treatment interruption, probably from accumulated 2-LTRc. It is a difficult task to estimate the exact impact of 2-LTRc as a substrate for integration under standard infection, i.e. WT infection in the absence of any anti-integrase drugs, due to their low representativeness compared to other viral DNA forms, especially DNA_L_ which represents the main DNA substrate form for integration. Here, we demonstrate that, under conditions where 2-LTRc accumulate in the infected cell, 2-LTRc constitutes a back-up molecule leading to DNA_L_ after IN-dependent cleavage at the palindromic junction. Such a cleavage is compatible with the integration of the HIV-1 genome into the host cell genome as well as with productive infection. Although DNA_L_ remains the substrate for integration, our data highlight that 2-LTRc should not be considered as a dead-end DNA product but, in contrast, could play a crucial role for viral resurgence in several circumstances such as pre-integration latency or RAL interruption.

## Conclusions

Our data demonstrate that RAL action is reversible and that unintegrated viral DNA can rescue viral replication after their integration in the host cell genome. Our results highly suggest that 2-LTR circles can be used as a reserve supply of genomes for proviral integration highlighting their potential role in the overall HIV-1 replication cycle.

## Methods

### Cells and viruses

HIV-1 stocks were prepared by transfecting 293 T cells with the various HIV-1 molecular clones derived from pNL4-3 (Additional file 1: Figure S2) [24]. Δenv viruses NLENG1-ES-IRES-WT and NLENG1-ES-IRES-D116N encode the WT integrase and catalytically inactive mutant D116N, respectively. Pseudotyping of Δenv viruses was performed by cotransfection of 293 T cells with a VSV-G plasmid. Virus preparations were treated with DNase I (Takara) in the presence of 10 mM MgCl_2_ at 37°C for 30 min and then untracentrifugation was performed (17,000 g for 1 hour). Aliquots were stored at-80°C. MT4 cells were culture in RPMI 1640. 293 T and HeLa-P4 cells were cultured in Dubelcco’s modified Eagle medium. All mediums were supplemented with 10% fetal calf serum, 100 units penicillin/ml and 100 μg streptomycin/ml (Invitrogen). PBMC were isolated from blood samples using Ficoll-Hypaque gradient centrifugation.

### HIV infectivity assay

The single-cycle titers of the virus on HeLa P4 cells were determined on HeLa CD4 LTR-LacZ cells in which the expression of β-galactosidase is inducible by the HIV transactivator protein Tat. Flow cytometry analysis was performed on a FACSCalibur flow cytometer.

### Viral infections

40 ng of p24^gag^ antigen per 10^6^ cells was used for infection. 3 hours after infection, cells were washed three times with PBS. Infections of PBMC were performed with 1 μg of p24^gag^ antigen per 10^6^ cells. When required, cells were treated in the presence of the RAL integrase inhibitor, with or without additional drugs (AZT, T-20, SAQ or Efavirenz).

### Quantifications of total HIV-1 DNA, 1-LTR circles (1-LTRc), 2-LTR circles (2-LTRc), linear DNA (DNA_L_) and integrated HIV-1 DNA (DNAi)

All DNA quantifications were performed by real-time PCR on a Light Cycler instrument. For each quantification, an equivalent of 200,000 cells was added in the PCR reaction. The sequences of the primers and probes used for real-time PCR for total HIV-1, 2-LTRc and integrated viral DNA quantifications have been described previously [34]. 1-LTRc and DNA_L_ were quantified according to [7]. Copy numbers of total HIV-1 DNA, 2-LTRc and DNA_L_ were determined from calibration curves obtained by amplifying pre-determined quantities of cloned DNA with matching sequences ranging from 10 to 10^5^ copies. DNAi quantification was performed by real-time Alu-LTR nested PCR, as previously described [34]. DNAi copy number was determined from a calibration curve obtained by concomitant two-stage PCR amplification of serial dilutions of a DNAi standard (from HeLa R7 Neo) mixed with uninfected cell DNA to yield 50,000 cell equivalents. The number of cell equivalents in sample DNA was calculated by amplifying the β-globin gene.

### Chromatin immunoprecipitation (ChIP)

ChIP assays were performed as previously described [35] at 24 and 72 hours post-infection. Briefly, 10^7^ infected cells were treated with 1% formaldehyde for 10 min at 37°C. Subsequent procedures were performed on ice with protease inhibitors. Cross-linked cells were harvested, washed with PBS, and lysed in lysis buffer (1% SDS, 10 mM EDTA, 50 mM Tris–HCl, pH = 8.1) for 10 min at 4°C. Chromatin was sonicated (six 10 s pulses at an amplitude of 30%). After centrifugation (14,000 g, 10 min, 4°C), the supernatant was diluted 10-fold with ChIP dilution buffer (0.01% SDS, 1% Triton X-100, 1.2 mM EDTA, 16.7 mM Tris–HCl, pH = 8.1, 167 mM NaCl). Diluted extracts were precleared with salmon sperm DNA-protein A-agarose beads (ChIP assay kit, Upstate). One tenth of the diluted extract was kept for quantitative PCR (input). Remaining extracts were incubated for 16 h at 4°C with 1 μg/ml of the specific antibody (from Upstate Biotechnology (anti-histone H3-06755) or from Santa Cruz (anti-HIV-1-integrase 1A1 sc-52418)) and then for 1 hour with salmon sperm DNA-protein A-agarose beads. Following extensive washing, bound DNA fragments were eluted. DNA was recovered by incubation for 4 hours at 65°C in elution buffer supplemented with 200 mM NaCl and incubated with proteinase-K (20 μg/ml) for 1 hour at 45°C. DNA was extracted before PCR quantification. The immunoprecipitated and input DNA were subjected to PCR quantification. Results are expressed as the fraction of immunoprecipitated DNA for each set of conditions.

### Western blot

3×10^6^ cells were lysed in RIPA buffer with protease inhibitors. 50 μg of protein were loaded on SDS-PAGE, transferred overnight to polyvinylidene difluoride (PVDF) membranes. The membranes were blocked in TBS-10% milk, incubated overnight at 4°C with primary antibody diluted in TBS-5% milk-0.05% Tween 20 (anti-integrase sc69721, Santa Cruz). Membranes were washed in TBS-0.1% Tween-20 and incubated for 1 h at room temperature with secondary antibody diluted in TBS-5% milk-0.05% Tween 20. Detection was performed by chemiluminescence (ECL).

### PIC preparation

Extract preparation were prepared as described previously with some modifications to allow the recovery of 2-LTRc complexed with viral and cellular proteins [31]. MT4 cells (2×10^7^) were infected with 20 μg of p24^Gag^ antigen of NLENG1-ES-IRES-D116N or NLENG1-ES-IRES-WT +/− 500 nM RAL in a total volume of 500 μl for 3 h at 37°C. Cells were then washed three times in 20 ml of PBS and resuspended in RPMI 1640 medium supplemented with 10% fetal calf serum and antibiotics at a final concentration of 2 × 10^5^ cells/ml. 72 hours post infection cells were harvested, washed twice with 25 ml of PBS and lysed in 3 cell pellet volumes of lysis buffer (20 mM Tris–HCl pH 8.0, 0.3 M KCl, 5 mM MgCl2, 10% (v/v) glycerol, 0.1% tween 20, 1 mM PMSF protease inhibitor cocktail from Sigma and RAL (500 nM) when required). Cell lysis was completed by two successive rounds of freeze-thaw, then incubated for 30 min at 4°C on rotating wheel. Two successive centrifugation steps at 16,000 g for 30 min at 4°C allowed complete removal of insoluble materials. The collected supernatant corresponding to soluble proteins within the cells was called whole cell extracts (WCE) and passed through 25G gauge needle attached on a 1 ml syringe. An aliquot was harvested and DNA was extracted as previously described. The remaining part was submitted to dialysis 3 and 6 hours at 37°C in a buffer allowing reaction (20 mM HEPES-KOH pH 7.4, 150 mM KCl, 1 mM MgCl2, 4% glycerol, and 1 mM DTT added just before starting the reaction). DNA was then submitted to proteinase K digestion (0.5 mg/ml) for 1 hour at 56°C and extracted with phenol:chloroform:isoamylalcohol 25:24:1. As the protocol does not allow a complete removal of the cellular genome, DNAi was quantified from the host cell genome. The remaining genomic DNA after PIC purification, quantified by quantitative PCR, represent 10% of the initial cellular DNA amount.

## Additional file

Additional file 1: Figure S1. Inhibition of integration by RAL. **Figure S2.** Viral constructs. **Figure S3.** Quantification of integrated viral DNA and influence of RAL. **Figure S4.** Synthesis of new infectious viral particles upon RAL removal. **Figure S5.** 2-LTR circles account for *de novo* integration and the resumption of viral replication after RAL removal. **Figure S6.** Detection of integrase by western blot analysis. **Figure S7.** 2-LTR circles account for de novo integration in primary cells after RAL removal. **Figure S8.** Reversibility of RAL in TC3 and TL3.4 cells. **Figure S9.** ChIP experiments performed on U3 and U5 regions.
