# Supplementary figures and images for: Integrase inhibitor reversal dynamics indicate unintegrated HIV-1 dna initiate de novo integration
Source: Retrovirology. 2015 Mar 12;12:24. doi: 10.1186/s12977-015-0153-9 (PMC4372172; doi:10.1186/s12977-015-0153-9)

Sup Fig1

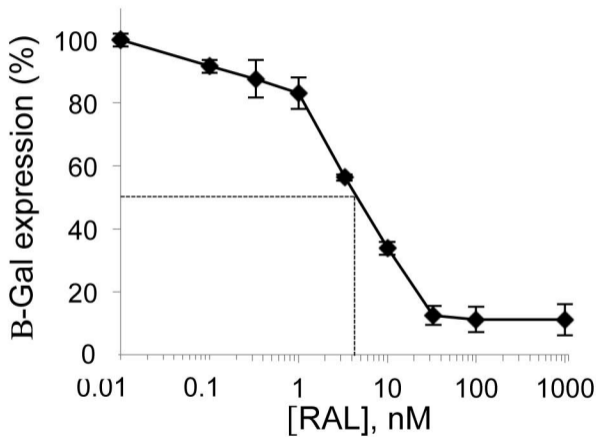

Sup Fig2

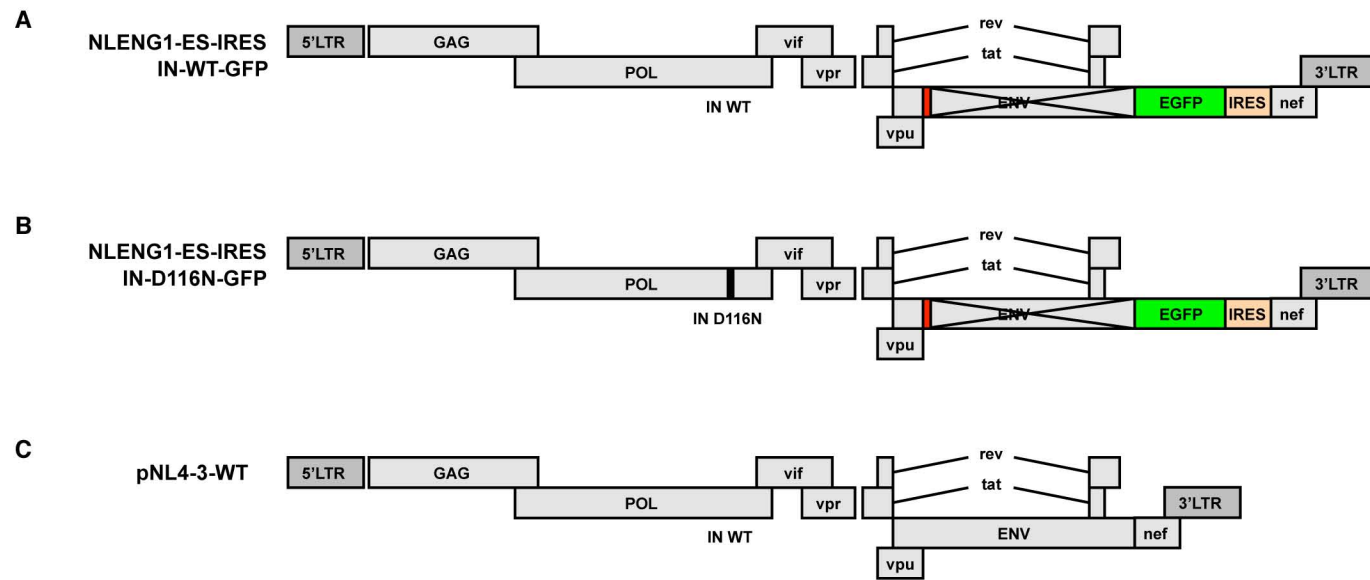

Sup Fig3

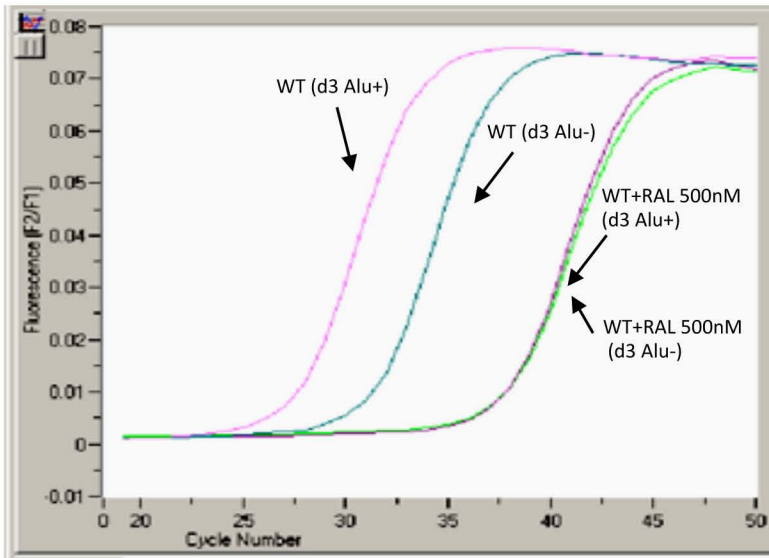

Sup Fig4

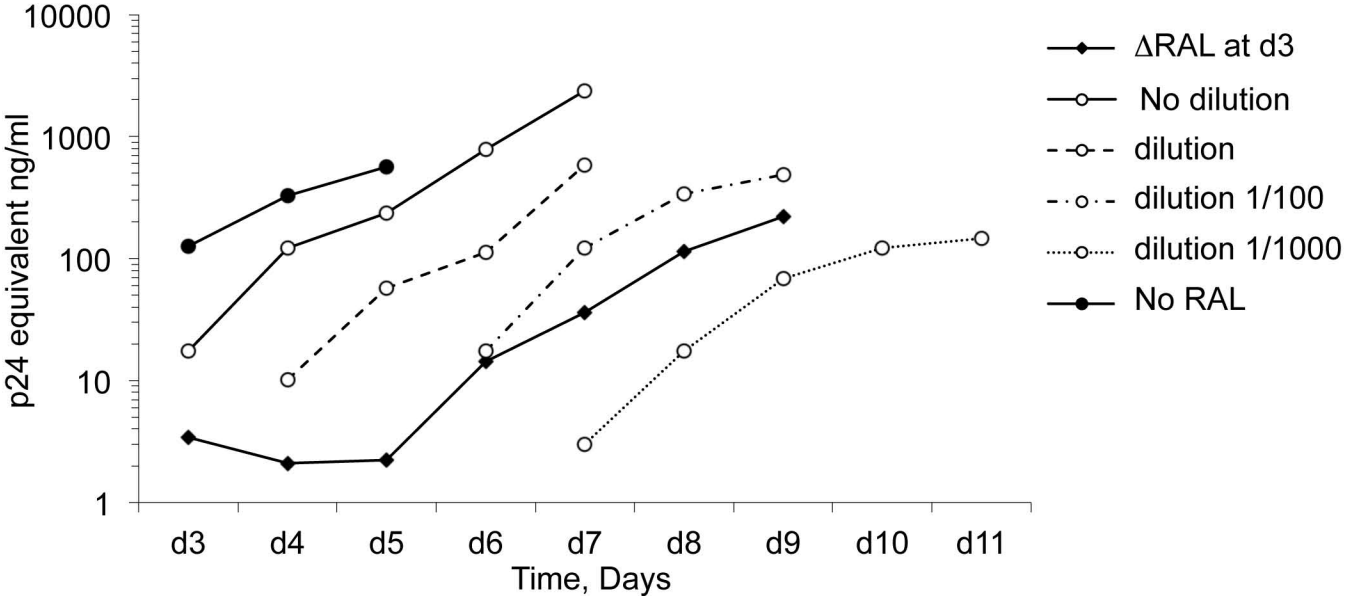

Sup Fig5

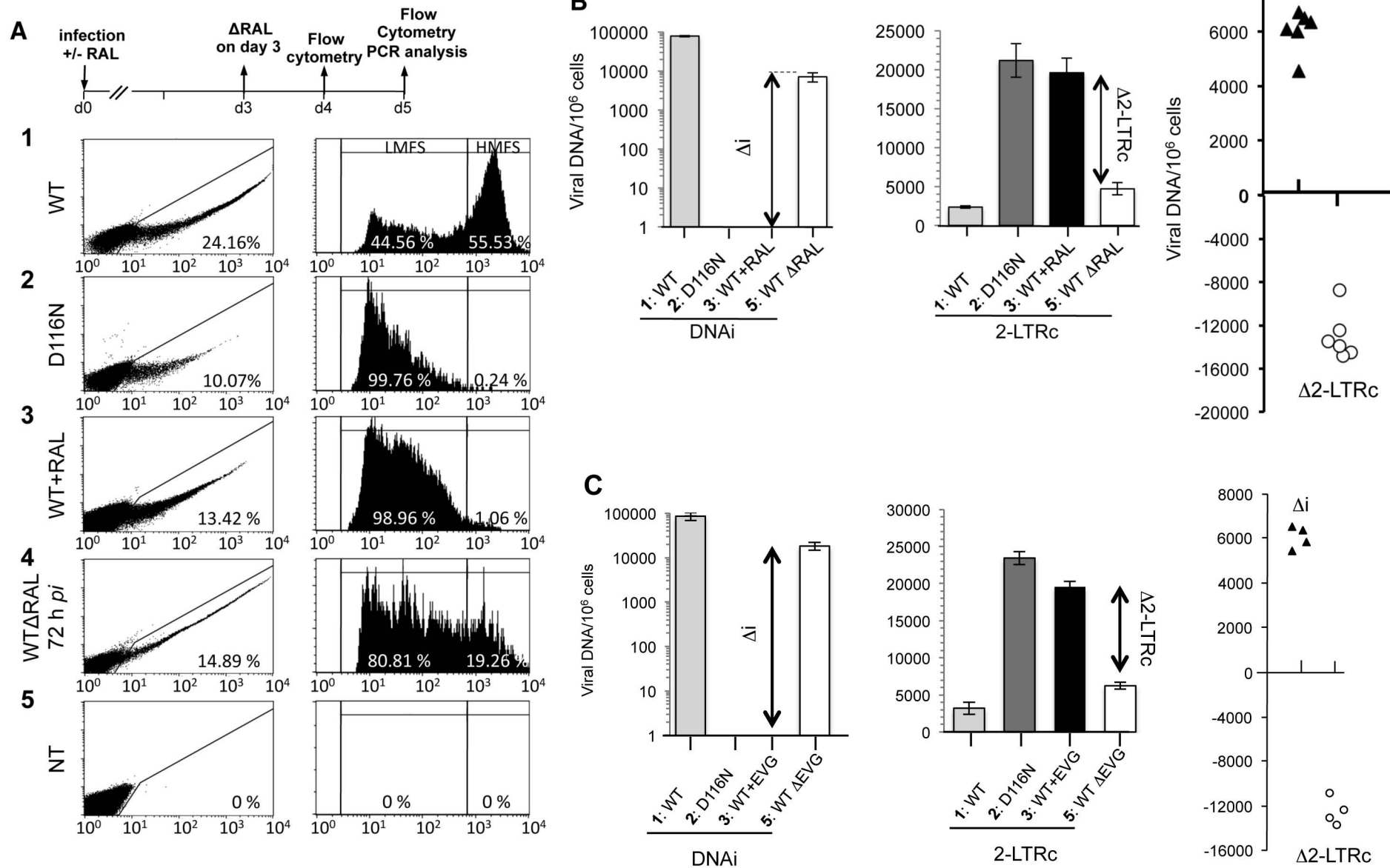

Sup Fig6

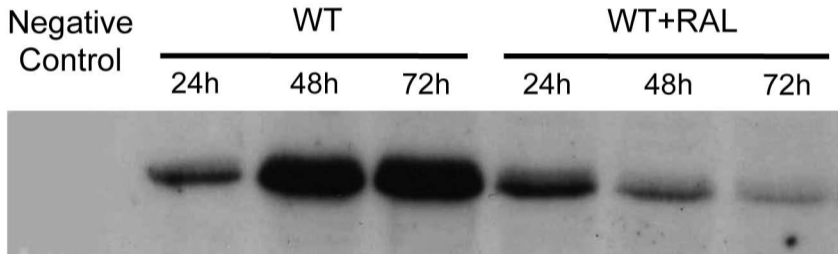



Sup Fig8

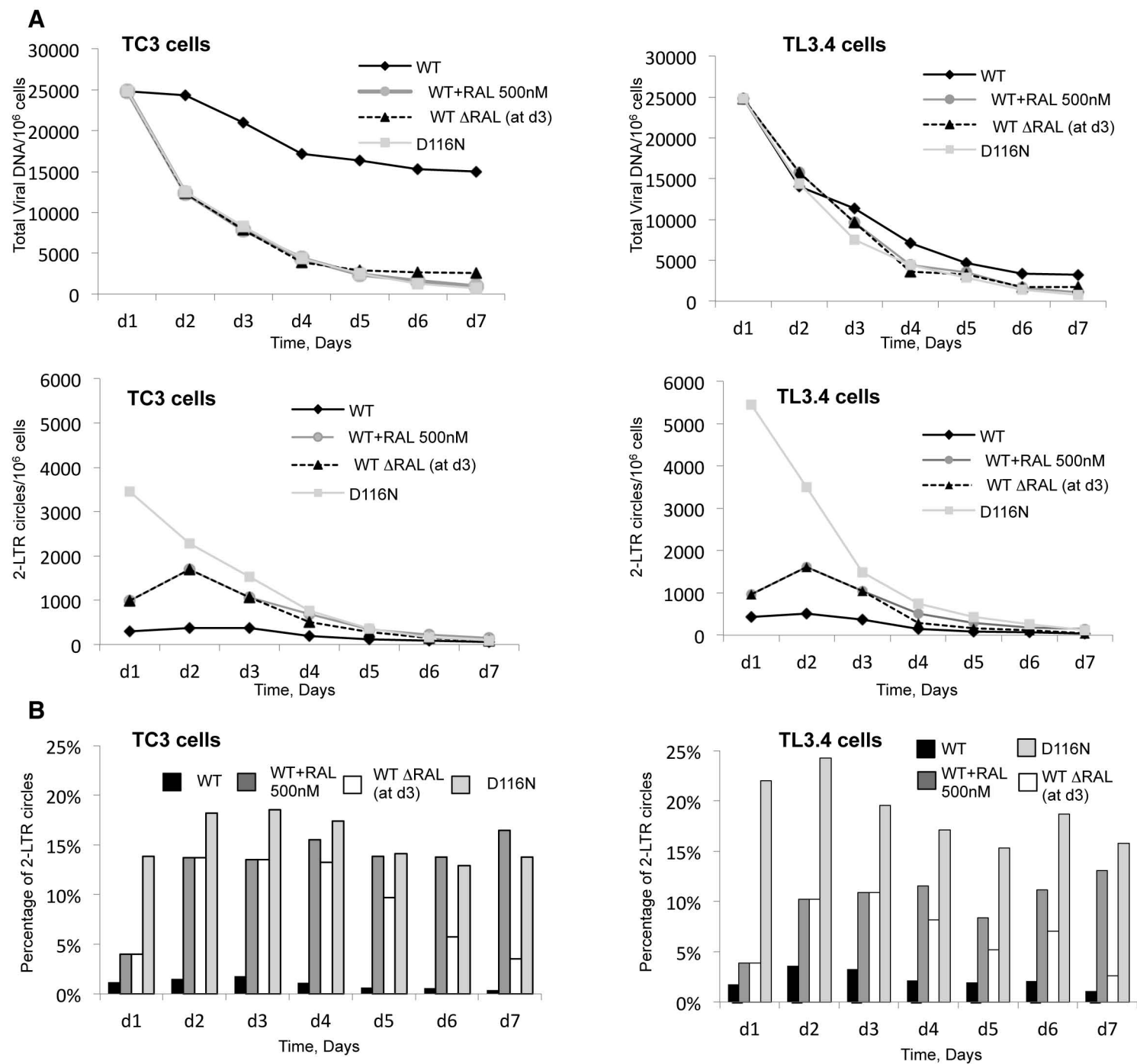

Sup Fig9

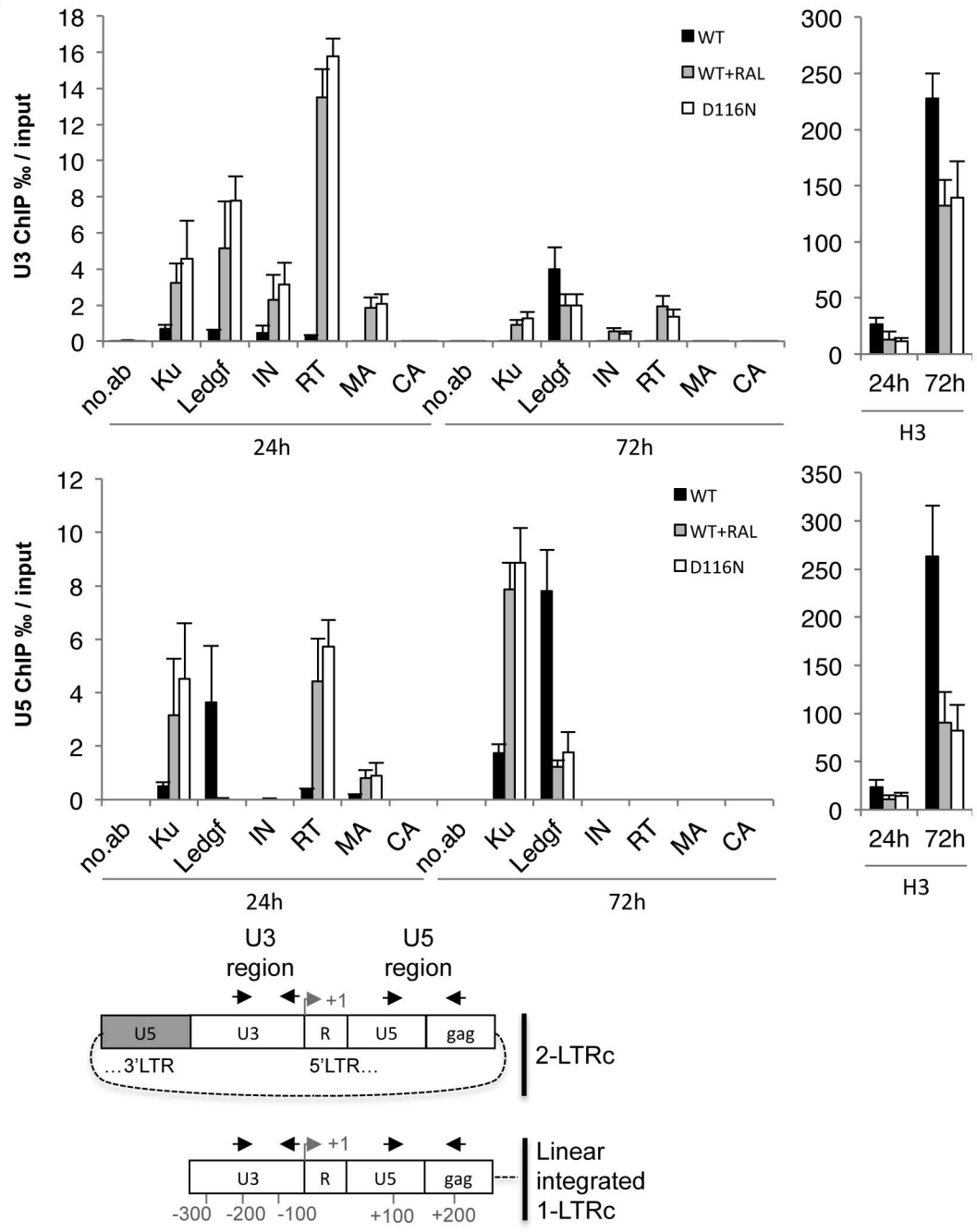

Supplement: Additional file 1: Figure S1. — Inhibition of integration by RAL. Figure S2. Viral constructs. Figure S3. Quantification of integrated viral DNA and influence of RAL. Figure S4. Synthesis of new infectious viral particles upon RAL removal. Figure S5. 2-LTR circles account for de novo integration and the resumption of viral replication after RAL removal. Figure S6. Detection of integrase by western blot analysis. Figure S7. 2-LTR circles account for de novo integration in primary cells after RAL removal. Figure S8. Reversibility of RAL in TC3 and TL3.4 cells. Figure S9. ChIP experiments performed on U3 and U5 regions. [file 12977_2015_153_MOESM1_ESM.pdf]
